# Supplementary material for: Efficacy and Safety of Native and Recombinant Zona Pellucida Immunocontraceptive Vaccines Formulated with Non-Freund’s Adjuvants in Donkeys
Source: Vaccines (Basel). 2022 Nov 24;10(12):1999. doi: 10.3390/vaccines10121999 (PMC9788400; doi:10.3390/vaccines10121999)
Supplement: Supplementary file 1 [file vaccines-10-01999-s001.zip › vaccines-1950390-supplementary.pdf]

Supplement

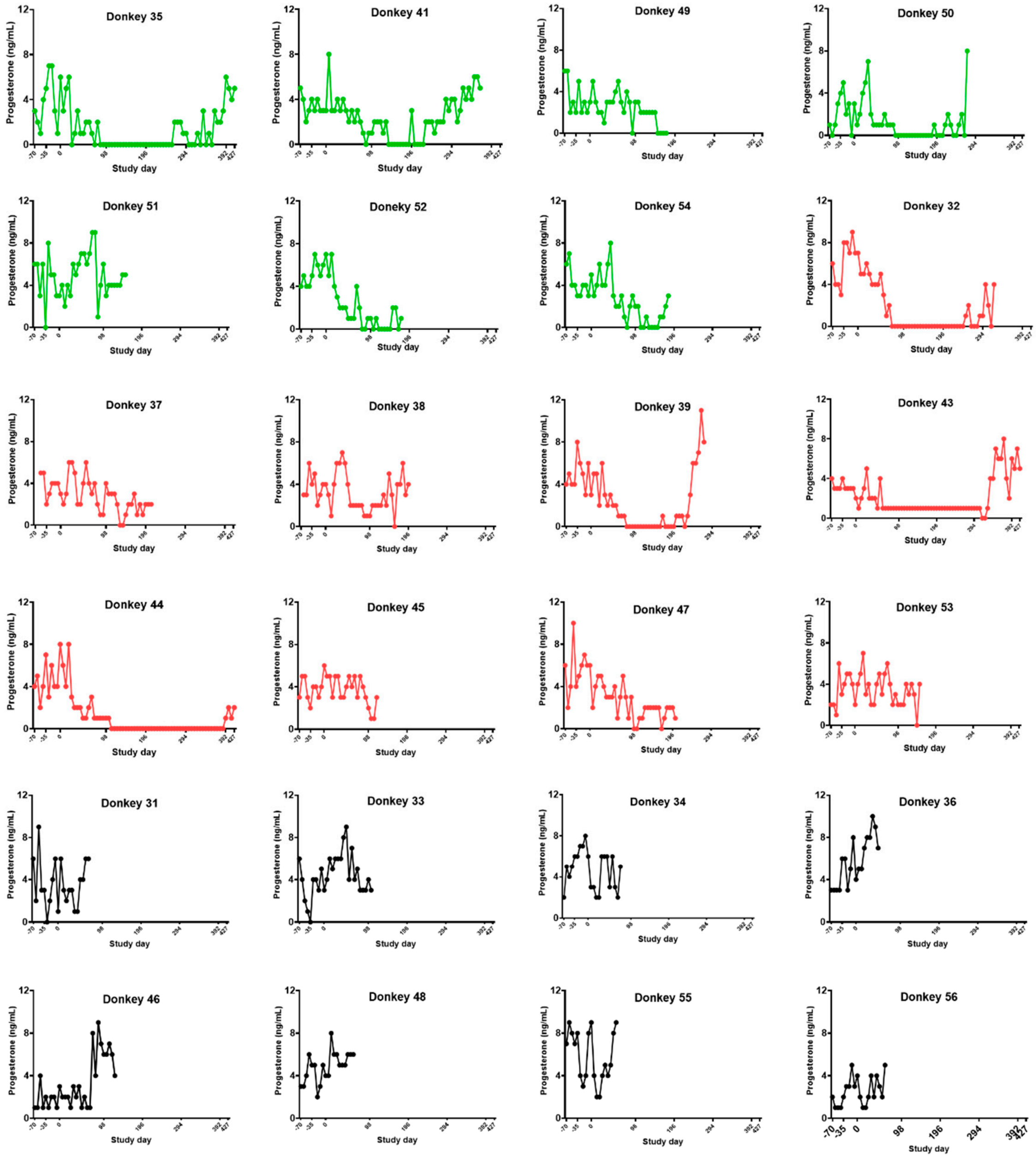

Figure S1. Progesterone concentration of jennies vaccinated with recombinant zona pellucida vaccine (reZP, green lines), native porcine zona pellucida (pZP, red lines), or placebo (Control, black lines) from Day -70 to the day of pregnancy diagnosis. Jennies were vaccinated with three doses of reZP on Days -70, -35 and 0. For both pZP and Control, the primary dose and the booster were administered on Days -35 and 0. Donkeys 35, 50, 52, 54 in reZP group, and 32, 39, 43, 44 in pZP group presented ovarian shutdown during the study.

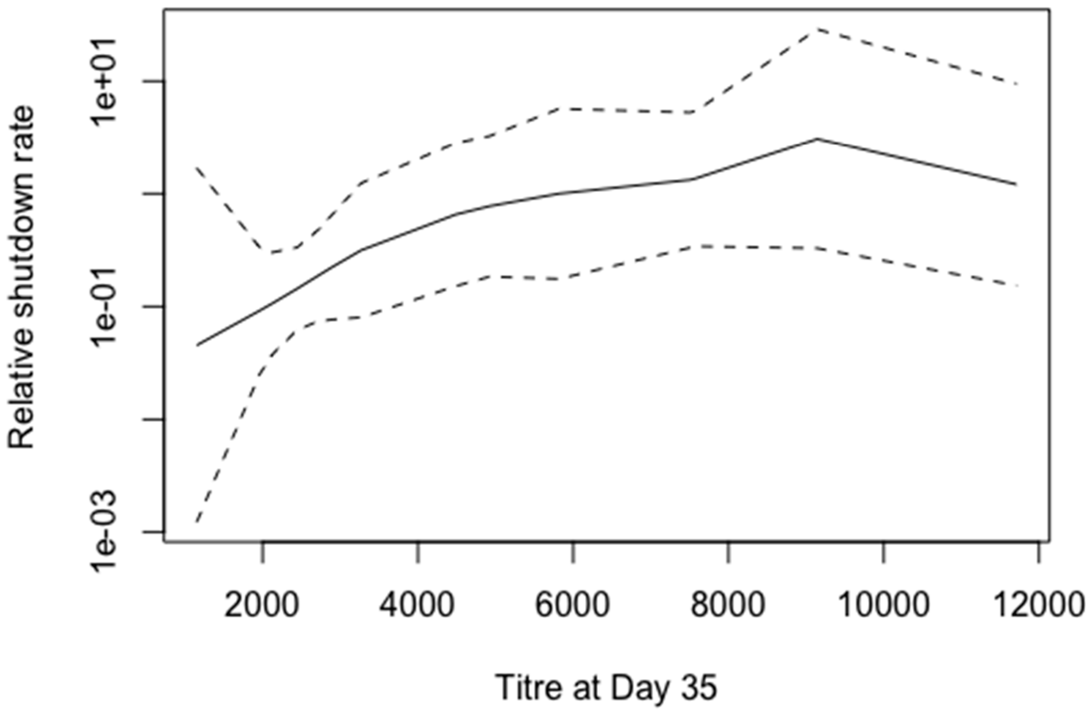

Figure S2. Effect of antibody titer at Day 35 (D35) on time to ovarian shutdown in the pZP and reZP groups combined. The solid line shows the shutdown rate relative to the mean D35 antibody titer as the reference. The dashed lines show the 95% confidence intervals.

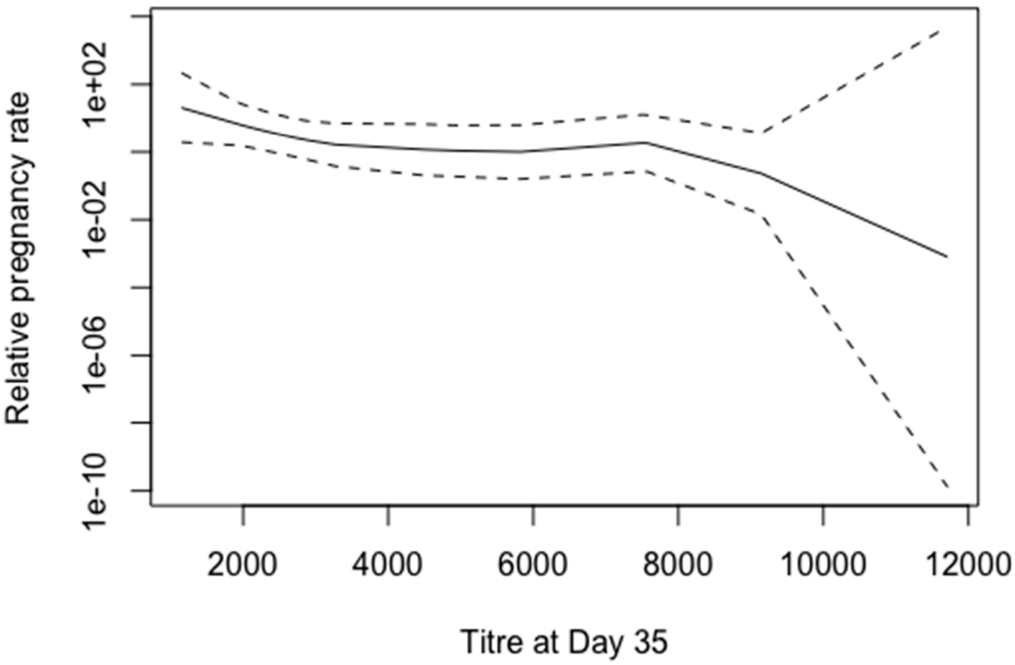

Figure S3. Effect of antibody titer at Day 35 (D35) on time to pregnancy in the pZP and reZP groups combined. The solid line shows the pregnancy rate relative to the mean D35 antibody titer as the reference. The dashed lines show the 95% confidence intervals.
